# Fermented Wheat Germ Extract Induced Cell Death and Enhanced Cytotoxicity of Cisplatin and 5-Fluorouracil on Human Hepatocellular Carcinoma Cells

**DOI:** 10.1155/2013/121725

**Published:** 2013-12-22

**Authors:** Cheng-Jeng Tai, Wen-Ching Wang, Chien-Kai Wang, Chih-Hsiung Wu, Mei-Due Yang, Yu-Jia Chang, Jiun-Yu Jian, Chen-Jei Tai

**Affiliations:** ^1^Division of Hematology and Oncology, Department of Internal Medicine, Taipei Medical University Hospital, Taipei 110, Taiwan; ^2^Department of Internal Medicine, School of Medicine, College of Medicine, Taipei Medical University, Taipei 110, Taiwan; ^3^Department of General Surgery, Chi Mei Medical Center, Tainan 710, Taiwan; ^4^Department of Chinese Medicine, Taipei Medical University Hospital, 252 Wu Hsing Street, Taipei 110, Taiwan; ^5^Department of Obstetrics and Gynecology, School of Medicine, College of Medicine, Taipei Medical University, Taipei 110, Taiwan; ^6^Department of Surgery, School of Medicine, College of Medicine, Taipei Medical University, Taipei 110, Taiwan; ^7^Center of Excellence for Cancer Research, Taipei Medical University, Taipei 110, Taiwan; ^8^Department of Surgery, Taipei Medical University-Shuang Ho Hospital, Taipei 235, Taiwan; ^9^Department of Surgery, China Medical University Hospital, Taichung 404, Taiwan; ^10^Graduate Institute of Clinical Medicine, College of Medicine, Taipei Medical University, Taipei 110, Taiwan; ^11^Department of Surgery, Taipei Medical University and Hospital, Taipei 110, Taiwan; ^12^Division of General Surgery, Department of Surgery, Taipei Medical University Hospital, Taipei Medical University, Taipei 110, Taiwan; ^13^Traditional Herbal Medicine Research Center, Taipei Medical University Hospital, Taipei Medical University, Taipei 110, Taiwan

## Abstract

Hepatocellular carcinoma (HCC) is one of the most common causes of cancer-related death worldwide. Due to the difficulties of early diagnosis, curative treatments are not available for most patients. Palliative treatments such as chemotherapy are often associated with low response rate, strong adverse effects and limited clinical benefits for patients. The alternative approaches such as fermented wheat germ extract (FWGE) with anti-tumor efficacy may provide improvements in the clinical outcome of current therapy for HCC. This study aimed to clarify antitumor efficacy of FWGE and the combination drug effect of FWGE with chemotherapeutic agents, cisplatin and 5-fluorouracil (5-Fu) in human HCC cells, HepG2, Hep3B, and HepJ5. The present study indicated that FWGE exhibited potential to suppress HepG2, Hep3B, and HepJ5 cells, with the half maximal inhibitory concentrations (IC_50_) of FWGE were 0.494, 0.371 and 1.524 mg/mL, respectively. FWGE also induced Poly (Adenosine diphosphate ribose) polymerase (PARP) associated cell death in Hep3B cells. Moreover, the FWGE treatment further enhanced the cytotoxicity of cisplatin in all tested HCC cells, and cytotoxicity of 5-Fu in a synergistic manner in HepJ5 cells. Collectively, the results identified the anti-tumor efficacy of FWGE in HCC cells and suggested that FWGE can be used as a supplement to effectively improve the tumor suppression efficiency of cisplatin and 5-Fu in HCC cells.

## 1. Introduction

Hepatocellular carcinoma (HCC) is one of the most common cancers worldwide and stands in the second place of cancer death [1], particularly in eastern Asia and sub-Saharan Africa [2]. The curative treatments for early-stage HCC are liver transplantation, resection, or local ablation, but these approaches are not viable for patients with advanced [2, 3] and the recurrence rate is as high as 50% at 2 years after operation [4]. Due to the difficulties in early diagnosis of HCC, about 70% of patients diagnosed with HCC are in advanced stage and unable to receive curative treatments [3]. In the development of alternative therapeutic approaches, palliative treatments such as chemoembolisation are suggested to show survival benefits in patients with advanced HCC [5, 6]. For example, although standard chemotherapeutic agents such as cisplatin and 5-Fluorouracil (5-Fu) administrated as systemic chemotherapy demonstrated no clinical benefit or improvement in survival [7, 8], hepatic arterial infusion and chemoembolisation with cisplatin and 5-Fu are considered potential therapeutic approaches for treating HCC [9]. In recent years, novel agents such as sorafenib also recommended for treating advance liver cancer [10]. Despite the development of therapeutic approaches for treating HCC, the mortality rate of patients with HCC still exceeds 90% worldwide [1]. Alternative treatments (e.g., components such as curcumin, resveratrol, silibinin isolated from natural products) that provide improvements in current clinical outcomes of HCC therapy are therefore in an urgent need [11].

The fermented wheat germ extract (FWGE), developed by Dr. Mate Hidvegi, is a nutrient supplement with medical value as demonstrated in a wide range of potential disease targets [12–14], including anti-tumor efficacy against many tumor types *in vitro* [15] and *in vivo* [16, 17]. Furthermore, some clinical studies also reported that the use of FWGE improved the overall survival in patients with colorectal cancer and skin melanoma. These data suggest that FWGE has the potential to provide benefits in cancer therapy [18, 19]. 2-methoxy benzoquinone and 2,6-dimethoxybenzene, the two major components of FWGE, are suggested to exert main biological properties of FWGE [14, 20]. Recent studies suggest that FWGE disrupts the anaerobic glycolysis and pentose cycle by targeting transketolase glucose-6-phosphate dehydrogenase, lactate dehydrogenase, and hexokinase [14, 21, 22], by which FWGE suppresses the allocation of precursors for DNA synthesis on tumor cells [13]. In the tumor cells of T-cell leukemia, FWGE treatment induced programmed cell death by interfering glycolysis and pentose cycle, resulting in cell cycle arrest and activation of the caspase-dependent Poly (Adenosine diphosphate ribose) polymerase (PARP) pathway [23]. The effects of FWGE combined with chemotherapeutic agents have been demonstrated on HCC, colorectal, ovarian, and breast cancer cells [16, 24, 25]. Results of these pioneer studies suggested that FWGE may enhance the cytotoxicity of cisplatin in ovarian cancer cells [24], and increase the efficacy of 5-Fu in colorectal cancer cells [15]. However, although the anti-proliferative effects of treatment with FWGE alone were demonstrated in human HCC and HepG2 cells [15], FWGE failed to enhance the cytotoxicity when combined with 5-Fu, Dacarbazine, or Adriblastina in the same cell lines [16]. Further clarification is required on the use of FWGE in combination with chemotherapeutic agents for HCC therapy.

Therefore, the aims of this study were to evaluate the anti-tumor effect of FWGE in human HCC cells, and to further clarify the effects of FWGE in combination with standard chemotherapeutic agents, cisplatin and 5-Fu. These data may provide a rational basis for the combined use of FWGE supplement and the development of therapeutic options in HCC therapy.

## 2. Materials and Methods

### 2.1. Cell Culture

Human hepatocellular carcinoma cell lines, HepG2, Hep3B, and HepJ5 were cultured in Dulbecco's modified Eagle's medium (Gibco, Grand Island, NY, USA) with 100 U/mL penicillin and 100 *μ*g/mL streptomycin (Invitrogen Life Technologies, Carlsbad, CA, USA) at 37°C in a 5% CO_2_ humidified incubator.

### 2.2. Cell Viability Assay and Microscopic Observation

HepG2, Hep3B, and HepJ5 cells were seeded into 96-well microplates at a density of 5 × 10^3^ cells per well overnight and then treated with various concentrations of fermented wheat germ extract (FWGE, brand name Avemar, American BioSciences Inc, Blauvelt, NY, USA) for 48 or 72 hr. Cell viability of tumor cells in this study was mainly determined by 3-(4,5-dimethylthiazol-2-yl)-2,5-diphenyltetrazolium bromide (MTT) assay and further confirmed by cell size measurements using a Scepter cell counter (Merck Millipore Billerica, MA, USA). Tumor cells were seeded into 24-well plates at a density of 3 × 10^4^ cells per well. After cultured overnight, cells were then exposed to serial dilutions of FWGE: 0, 0.25, 0.5 mg/mL for HepG2 cells, 0, 0.2, 0.4 mg/mL for Hep3B cells, and 0, 0.5, 1 mg/mL for HepJ2 cells. Cells were harvested by trypsinization 72 h after adding FWGE and cell number was counted by a Scepter cell counter.

To investigate the influence of FWGE combined with cisplatin and 5-fluorouracil (5-Fu, both agents were purchased from Sigma-Aldrich, St Louis, MO, USA), HepG2, Hep3B and HepJ5 cells were treated with various concentrations of cisplatin and 5-Fu with 0.5, 0.25 and 1 mg/mL FWGE. After 72 hr, cell viability was determined by MTT assay and morphology was observed with a Nikon Eclipse TS100 optical microscope (Nikon Instruments, Melville, NY, USA) and photographed at 100x magnification.

### 2.3. Western Blotting Analysis

HepG2, Hep3B, and HepJ5 cells (5 × 10^5^ cells per dish) were seeded in 6 cm dishes overnight. After incubation with various concentrations of FWGE (as indicated), cells were harvested by RIPA buffer (150 mM NaCl, 50 mM pH 7.5 Tris-HCL, 1% NP-40, 0.5% deoxycholate, 0.1% SDS, 1 mM PMSF, 10 *μ*g/mL leupeptin, and 100 *μ*g/mL aprotinin). The total protein concentrations from whole cell extracts were determined by a Bio-Rad protein assay kit (Bio-Rad Laboratories, Hercules, CA, USA). Each cell extract was then equalized to 30 *μ*g and separated using 12% sodium dodecyl sulfate polyacrylamide gel electrophoresis. The proteins were transferred onto a polyvinylidene fluoride membrane (Pall Corp., Port Washington, NY, USA) and probed with the primary antibodies, PARP (1 : 1,000, Cell Signaling Technology, Danvers, MA, USA), and glyceraldehyde 3-phosphate dehydrogenase (GAPDH, 1 : 10,000, Abfroniter, Seoul, Korea), followed by donkey antirabbit horseradish peroxidase conjugated secondary antibody (1 : 10,000, Santa Cruz Biotechnology, Santa Cruz, CA, USA). Immunoreactivity was then detected with a electrochemiluminescence western blotting detection kit (WesternBright, Advabsta, Menlo Park, CA, USA).

### 2.4. Statistical Analysis

Data from cell viability and semiquantitative western blotting analysis were presented as mean ± stand derivation (SD). Statistical significance was analyzed one-way ANOVA when examining the dose dependent effect. Statistical analysis was performed by SPSS (SPSS Inc, Chicago, IL, USA).

CalcuSyn software (Biosoft, Cambridge, UK) was used for the statistical analysis of the half maximal inhibitory concentration (IC_50_) determined by MTT assay and for the combined effects of FWGE with chemotherapeutic drugs. Statistical analysis of the combined drug effects with the CalcuSyn software is based on the median-effect method and evaluated by the combination index (CI) value [26], which is a useful tool for identifying synergistic, additive and antagonistic effects between components on cancer cells [27, 28].

## 3. Results

### 3.1. FWGE Treatment Induced Cell Death in Human Hepatocellular Carcinoma Cells

**Figure 1:**
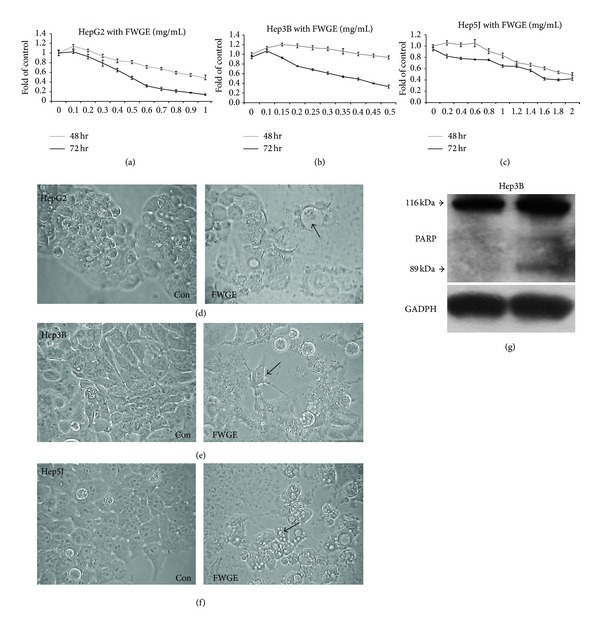
FWGE treatment inhibited cell growth of human HCC cells, HepG2, 3B, and J5. (a) to (c) cells were treated with a serial dilutions of FWGE in HepG2, 3B, and J5 cells for 48 or 72 hr. Cell viability was analyzed by MTT assay. Experiments were performed in triplicate at least and data were shown as mean ± SD. All cell lines showed dose-dependent effects at 72 hr after FWGE treatment in the MTT assay (one-way ANOVA, *P* < 0.01). (d) to (f) where FWGE-induced morphological changes in HepG2, Hep3B, and HepJ5 cells treated with 0.4, 0.25 and 1 mg/mL for 72 hr. Arrows indicated morphological changes in FWGE treated cells. (g) Western blotting analysis of PARP for Hep3B cells treated with 0.25 mg/mL FWGE for 72 hr. PARP was 116 KDa and cleaved PARP was 89 kDa.

**Figure 2:**
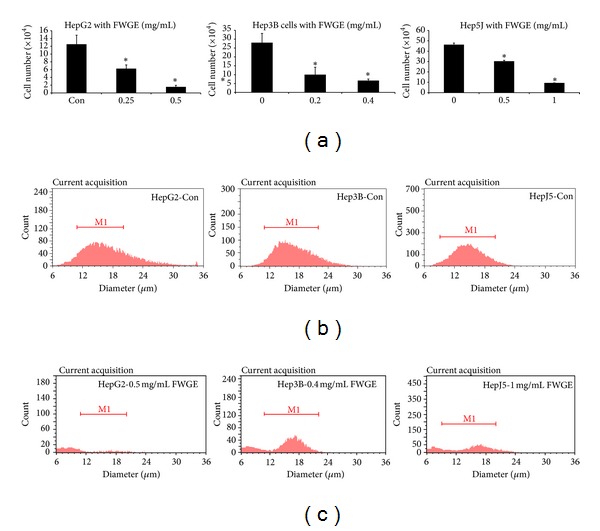
FWGE-induced cell death in HCC cells. (a) HepG2, Hep3B, and HepJ5 cells were treated by FWGE for 72 hr. Survival cells were determined by size measurements for the cells sized between 10 to 22 *μ*m. (a) Survival cell number of FWGE treated HepG2, Hep3B, and HepJ5 cells after 72 hr. All cell lines showed dose-dependent effects following FWGE treatment (one-way ANOVA, *P* < 0.01 on HepG2 and Hep3B cells and *P* < 0.05 on HepJ5 cells). Experiments were performed in triplicate at least and data were shown as mean ± SD. (b) Cell size distribution of cells in control medium or (c) FWGE (0.5, 0.4 and 1.0 mg/mL in HepG2, Hep3B, and HepJ5 cells for 72 hr) treated cells.

In present study, antiproliferative activity of a 48 or 72 hr continuous exposure to various concentrations of FWGE was evaluated in three human HCC cells, HepG2, hep3B and HepJ5 cells. As shown in Figures 1(a) to 1(c), anti-proliferative effects of FWGE in three tested tumor cells demonstrated a dose-dependent manner. In HepG2 and Hep3B cells, FWGE treatment for 72 hr resulted in a greater inhibitory effect on cell growth than 48 hr treatment. In contrast, FWGE treatment exerted a similar growth inhibitory effect in HepJ5 cells for 48 and 72 hrs. IC_50_ of FWGE were 0.494, 0.371 and 1.524 mg/mL for HepG2, Hep3B, and HepJ5 cells, respectively, suggesting that HepG2 and Hep 3B cells were more sensitive to FWGE treatment than HepJ5 cells (Table 1). Morphological changes observed in FWGE treated cells suggested that FWGE induced cell death rather than cell growth inhibition (Figures 1(d) to 1(e)). FWGE treatment also led to cell shrinkage in HepG2 and Hep3B cells and the presence of apoptosis body like vesicles around shrinking cells (Figures 1(d) and 1(e)), whereas FWGE increased the formation of lipid droplet like vesicles in HepJ5 cells (Figure 1(f)). Cell viability determined by MTT assay is based on the measurement of metabolic activity of mitochondrial oxidoreductases in survival cells, but may be biased when the metabolic activity of survival cells was disrupted by specific stress on mitochondria [29]. To further confirm the anti-proliferative effects of FWGE on tested tumor cells, cell size distribution was determined. Cell number counted by a Scepter cell counter was based on Coulter principle of impedance-based particle detection [30]. Cells with a diameter ranged between 10 to 22 *μ*m were counted as survival cells. In Figure 2(a), survival cell numbers of HepG2, 3B, and J5 cells were significantly decreased in a dose-dependent manner after FWGE treatment for 72 hr. Moreover, FWGE treatment resulted in more cells with a much smaller size (6 to 8 *μ*m) suggesting the accumulation of cell debris from dead cells (Figures 2(b) and 2(c)). The findings of Western blotting analysis also indicated activation of PARP in Hep3B cells treated with 0.25 mg/mL FWGE for 72 hr. These results together with morphological changes observed in FWGE treated cells indicated that FWGE was likely to trigger programmed cell death rather than inhibit proliferation of tumor cells.

Interestingly, cell viability determined by MTT assay was higher than counted by a Scepter cell counter. For example, the viability of HepG2 cells following 0.5 mg/mL FWGE treatment was 85% as determined by MTT assay, whereas the result counted by a Scepter cell counter was 50%. Similar results were observed in Hep3B and J5 cells. Hep3B and HepJ5 cells exposed to 0.2 and 0.5 mg/mL FWGE revealed 75% and 77% cell viability by MTT assay but were 35% and 65% as determined a Scepter cell counting. Together these results suggested that MTT assay may underestimate the anti-tumor efficiency of FWGE on HCC cells.

### 3.2. FWGE Enhanced Cytotoxicity of Chemotherapeutic Drugs on Human HCC Cells

**Figure 3:**
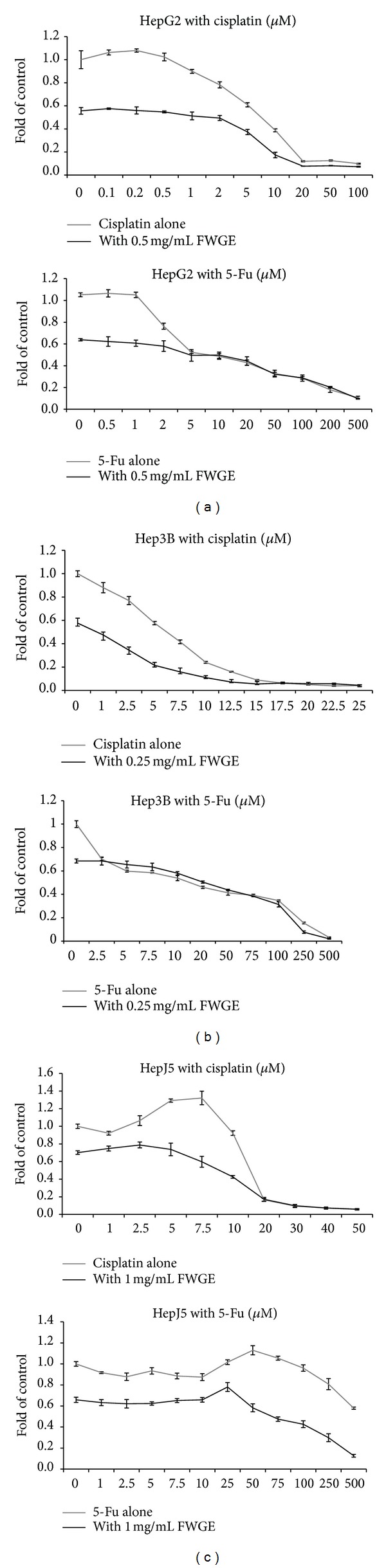
Combination of FWGE with cisplatin or 5-Fu in HepG2, Hep3B, and HepJ5 cells. (a) HepG2; (b) Hep3B; (c) HepJ5 cells. Cells were treated serial dilutions of cisplatin or 5-Fu alone or in combination of FWGE (0.5, 0.25 and 1 mg/mL FWGE on HepG2, Hep3B, and HepJ5 cells, resp.) for 72 hr. Cell viability was analyzed by MTT assay. Experiments were performed in triplicate at least and data were shown as mean ± SD.

For evaluating the effects of combination of FWGE and chemotherapeutic drugs on HCC cells, HepG2, 3B, and J5 cells were treated with various doses of cisplatin or 5-Fu with 0.5, 0.25 and 1 mg/mL FWGE, respectively. The FWGE doses used for each of cell lines were approximately 60% inhibition achieved in previous results (Figure 1). The use of IC_60_ of FWGE was to avoid over suppression of cell viability and resulted in the difficulty of evaluation on combination effect of FWGE and chemotherapeutic drugs. The viability of tumor cells exposed to cisplatin or 5-Fu alone or combinations with FWGE was shown in Figure 3. Results suggested that FWGE further decreased cell viability of HepG2 and Hep3B cells in the presence of cisplatin, and HepJ5 in the presence of 5-Fu. Subsequent to treatment, FWGE decreased the IC_50_ of cisplatin from 6.843 to 1.049, 4.436 to 1.111, and 15.785 to 6.021 *μ*M in hepG2, Hep3B, and HepJ5 cells, respectively. In 5-Fu treated cells, co-treatment with FWGE only slightly decreased the IC_50_ of 5-Fu from 5.237 to 4.591 in HepG2 but not Hep3B cells (Table 1). Although HepJ5 was relatively resistant to 5-Fu treatment compared with HepG2 and Hep3B cells, FWGE combined with 5-Fu still led to a greater inhibition for HepJ5 cells (Figure 3 and Table 1). These results together suggested that cytotoxicity of cisplatin and 5-Fu may be further enhanced by co-treatment of FWGE. Combination index (CI) analyzed by Calcusyn software may help to identify the combination effects of FWGE with cisplain and 5-Fu on HCC cells. By which, synergistic, additive, and antagonistic effects were indicated by CI values of <1, =1, and >1. As shown in Table 2, FWGE treatment was found to result in additive effect in cells treated with 1 to 2 *μ*M of cisplatin (CI closed to 1), and synergic effect in 5 to 20 *μ*M of cisplatin (CI < 1) for HepG2 cells. Whereas synergic effects in 1 to 15 and 5 to 30 *μ*M cisplatin were observed in Hep3B and HepJ5 cells, respectively. FWGE treatment also showed the synergic effect with 1 to 50 and 50 to 250 *μ*M 5-Fu in HepG2 and HepJ5 cells. Antagonistic effect (CI > 1) was observed in Hep3B cells, but cell viability was similar in various tumor cells treated with 5-Fu alone and combined with FWGE (Figure 3(b)). According to CI analysis, cotreatment with FWGE may enhance cytotoxicity of cisplatin and 5-Fu, depending on cell types and administrated doses of chemotherapeutic agents in human HCC cells.

## 4. Discussion

The present study demonstrated that FWGE exhibited an effective anti-tumor activity against human HCC cells, particularly in HepG2 and Hep3B cells with IC_50_ values of 0.494 and 0.371 mg/mL. In contrast, HepJ5 was more resistant to FWGE treatment with an IC_50_ of 1.524 mg/mL. Our findings were comparable to previous studies indicating an IC_50_ of FWGE treatment for 96 hr was 0.33 mg/mL in HepG2 cells. Interestingly, cell viability determined by MTT assay significantly overestimated the survival of FWGE treated HCC cells compared with the measurements of cell size distribution, particularly for HepG2 (85% versus 56% of cell viability at 0.25 mg/mL FWGE) and Hep3B (75% versus 35% cell viability at 0.2 mg/mL). Since the cell viability measured by MTT assay depending on the colorimetrics of purple formazan converted by mitochondrial dehydrogenases [31], the FWGE pentose phosphate pathway disrupted by FWGE might lead to altered metabolic activity of mitochondria in HCC cells and resulted in an overestimated measurement of optic density. In HepJ5 cells, the difference between MTT assay and cell size distribution was smaller in HepG2 and Hep3B cells, suggesting that the FWGE treatment may cause less mitochondrial metabolic disruption in HepJ5 cells. Tung and colleagues suggested that hypoxia inducible factor 1-alpha (HIF 1-*α*) is overexpressed in HepJ5 cells and plays a protective role in mitochondrial mediated apoptosis [32]. This biological feature may help HepJ5 cells to evade destruction and death induced by FWGE.

FWGE treatment also resulted in obvious morphological changes in three HCC cell lines. In both HepG2 and Hep3B cells, cell shrinkage and formation of apoptotic body-like vesicles occurred after FWGE treatment, whereas lipid-droplet like vesicles was shown in cytoplasm of HepJ5. Cell size distribution of HCC cells ranged from 10 to 22 *μ*m and shifted to 6~8 *μ*m in cells treated with FWGE, suggesting that the HCC cells were broken down into fragments. These results together suggested FWGE induced cell death of HCC cells. Morphological changes observed in Hep3B and G2 cells indicated that apoptosis might be involved in FWGE-induced cell death. Cleavage of PARP in Hep3B cells further confirmed this observation and suggested that FWGE induced HCC cell death was associated with PARP involved programmed cell death. Since FWGE induced cell death on T-cell leukemia cells was involved in caspase-PARP pathway [23], PARP activation is likely to play a critical role in FWGE induced cell death in tumor cells. On the other hand, the specific morphological change and formation of lipid-droplet like vesicles following FWGE treatment indicated a cell-specific response to FWGE treatment in HepJ5 cells. This lipid-droplet like vesicles was similar to autophagosome and therefore raised a doubt that a protective autophagic process was activated in HepJ5 cells to evade destruction and subsequent cell death induced by FWGE. Since the autophagy is a double-edge sword as a programmed cell death process or a preventing cell death from lethal stress, particularly in cancer cells [33], whether FWGE induces autophagy and the role of autophagy in HepJ5 cells may warrant further investigation.

FWGE is considered as a safe nutrient supplement in recommended dosage for medical purposes including cancer patients without observed adverse effects [14], though the clinical significance of FWGE as a drug component remains to be verified by more well-designed clinical trials [34]. FWGE was used as an adjuvant treatment with chemotherapy and radiotherapy for colorectal cancer and skin melanoma in some clinical studies [18, 19]. Therefore, the effects of FWGE combined with chemotherapeutic agents, cisplatin and 5-Fu, were tested in the present study to clarify the clinical potential of FWGE for HCC treatment. In HepG2 and Hep3B cells, cisplatin induced cytotoxicity was greatly enhanced by FWGE and resulted in decrease of the IC_50_ dosage (Table 1). Although IC_50_ was not available in HepJ5 cells, FWGE treatment still enhanced cisplatin-induced cytotoxicity in Hepj5, HepG2, and Hep3B cells. The CI analysis suggested that the effects of FWGE enhanced cisplatin-induced cytotoxicity was synergistic at 5–20, 1–15 and 5–30 *μ*M for HepG2, Hep3B and HepJ5 cells, respectively. These data were coincident with the observation in human ovarian cancer cells treated with FWGE and cisplatin [24]. On the other hand, FWGE co-treatment with 5-Fu enhanced the cytotoxicity of 5-Fu at IC_50_ dosage in HepJ5 cells, but not HebG2 and Hep3B cells. CI analysis also indicated a synergistic effect of FWGE on 5-Fu in HepJ5 cells. Due to the high similarity of cell viability curves after treatment with FWGE and 5-Fu in HepG2 and Hep3B cells, CI analysis was unable to clearly identify the combination effect of FWGE and 5-Fu in these cell lines. Nevertheless, FWGE with cisplain or 5-Fu demonstrated no antagonistic effect in tested HCC cells. Since FWGE activated programmed cell death in HCC cells, whether the pathway of FWGE related cell death also interacts with cisplatin or 5-Fu induced apoptosis requires further investigation.

## 5. Conclusion

**Figure 4:**
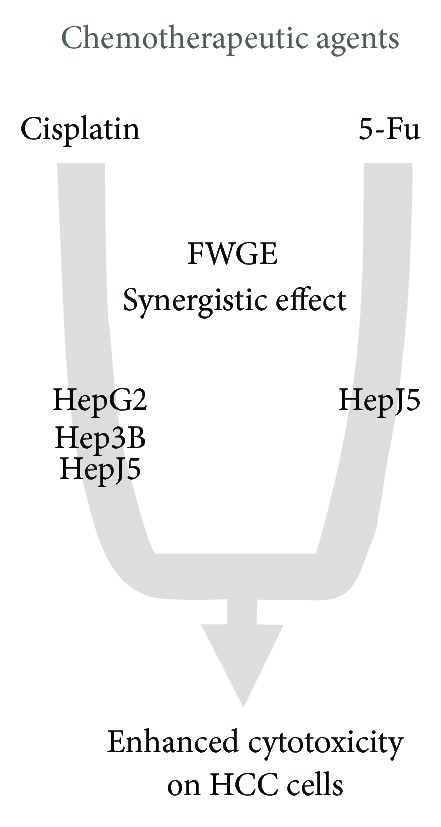
Conclusion on the combination effect of FWGE with chemotherapeutic agents, cisplain, and 5-Fu in HCC cells.

FWGE exhibited an anti-tumor efficacy and induced cell death in human HCC cells, including HepG2, Hep3B, and HepJ5 cells. In combination with chemotherapeutic agents, cisplatin and 5-Fu, FWGE showed no antagonistic effect, and eventually enhanced cisplatin-induced cytotoxicity in HepG2, Hep3B, and HepJ5 cells, as well as 5-Fu-induced cytotoxicity in HepJ5 cells. The combination effect of cisplatin and 5-Fu were summarized in Figure 4. In conclusion, the results of this study suggest that FWGE is a potential adjuvant treatment to improve tumor suppression efficiency, particularly in treatment regimens using cisplatin and 5-Fu for patients with HCC.

## Figures and Tables

**Table 1:** The half maximal inhibitory concentration (IC_50_) of FWGE calculated in human HCC cells. IC_50_ were determined following treatment with FWGE alone or combined with chemotherapeutic drugs by MTT assay after 72 hr.

	HepG2		Hep3B		HepJ5	
FWGE (mg/mL)	0.494		0.371		1.524	

	Combination of FWGE (mg/mL)					
	0	0.5	0	0.25	0	1

Cisplatin (*μ*M)	6.843	1.049	4.436	1.111	15.785	6.021
5-Fu (*μ*M)	5.237	4.591	14.427	15.306	>500	70.571

**Table 2:** Analysis of combination effects of FWGE with cisplatin or 5-Fu in human HCC cells. Cell viability of FWGE treated cells with cisplain or 5-Fu for 72 hr were determined by MTT assay. Combination index (CI) was obtained by using Calcusyn software. CI < 1 indicated a synergistic effect, CI = 1 indicated an additive effect, and CI > 1 indicated an antagonistic effect.

	HepG2		Hep3B		HepJ5	
	Dose	CI	Dose	CI	Dose	CI
Combination of FWGE (mg/mL)	0.5		0.25		1	

Cisplatin (*μ*M)	1	1.03	1	0.41	5	0.75
	2	1.05	2.5	0.51	7.5	0.64
	5	0.71	5	0.61	10	0.54
	10	0.41	7.5	0.71	20	0.52
	20	0.41	10	0.71	30	0.58
	50	1.03	12.5	0.68		
			15	0.69		

5-Fu (*μ*M)	1	0.41	5	0.96	50	0.32
	2	0.34	7.5	1.25	75	0.35
	5	0.25	10	1.16	100	0.02
	10	0.41	20	1.44	250	0.01
	20	0.57	50	2.35		
	50	0.85	75	2.49		
	100	1.47	100	2.05		

## References

[1] Jemal A, Bray F, Center MM, Ferlay J, Ward E, Forman D (2011). Global cancer statistics. *CA: Cancer Journal for Clinicians*.

[2] El-Serag HB (2011). Hepatocellular carcinoma. *The New England Journal of Medicine*.

[3] Bruix J, Llovet JM (2009). Major achievements in hepatocellular carcinoma. *The Lancet*.

[4] Yamamoto M, Arii S, Sugahara K, Tobe T (1996). Adjuvant oral chemotherapy to prevent recurrence after curative resection for hepatocellular carcinoma. *The British Journal of Surgery*.

[5] Llovet JM, Bruix J (2003). Systematic review of randomized trials for unresectable hepatocellular carcinoma: chemoembolization improves survival. *Hepatology*.

[6] Braillon A (2012). Hepatocellular carcinoma. *The Lancet*.

[7] Llovet JM, Burroughs A, Bruix J (2003). Hepatocellular carcinoma. *The Lancet*.

[8] Carr BI (2004). Hepatocellular carcinoma: current management and future trends. *Gastroenterology*.

[9] Ishikawa T (2009). Future perspectives on the treatment of hepatocellular carcinoma with cisplatin. *World Journal of Hepatology*.

[10] Jelic S, Sotiropoulos GC (2010). Hepatocellular carcinoma: ESMO clinical practice guidelines for diagnosis, treatment and follow-up. *Annals of Oncology*.

[11] Li Y, Martin RC (2011). Herbal medicine and hepatocellular carcinoma: applications and challenges. *Evidence-Based Complementary and Alternative Medicine*.

[12] Iyer A, Brown L (2011). Fermented wheat germ extract (Avemar) in the treatment of cardiac remodeling and metabolic symptoms in rats. *Evidence-Based Complementary and Alternative Medicine*.

[13] Boros LG, Nichelatti M, Shoenfeld Y (2005). Fermented wheat germ extract (Avemar) in the treatment of cancer and autoimmune diseases. *Annals of the New York Academy of Sciences*.

[14] Johanning GL, Wang-Johanning F (2007). Efficacy of a medical nutriment in the treatment of cancer. *Alternative Therapies in Health and Medicine*.

[15] Mueller T, Jordan K, Voigt W (2011). Promising cytotoxic activity profile of fermented wheat germ extract (Avemar) in human cancer cell lines. *Journal of Experimental & Clinical Cancer Research*.

[16] Szende B, Marcsek Z, Kocsis Z, Tompa A (2004). Effect of simultaneous administration of Avemar and cytostatic drugs on viability of cell cultures, growth of experimental tumors, and survival of tumor-bearing mice. *Cancer Biotherapy and Radiopharmaceuticals*.

[17] Zalatnai A, Lapis K, Szende B (2001). Wheat germ extract inhibits experimental colon carcinogenesis in F-344 rats. *Carcinogenesis*.

[18] Jakab F, Shoenfeld Y, Balogh Á (2003). A medical nutriment has supportive value in the treatment of colorectal cancer. *The British Journal of Cancer*.

[19] Demidov LV, Manziuk LV, Kharkevitch GY, Pirogova NA, Artamonova EV (2008). Adjuvant fermented wheat germ extract (Avemar) nutraceutical improves survival of high-risk skin melanoma patients: a randomized, pilot, phase II clinical study with a 7-year follow-up. *Cancer Biotherapy and Radiopharmaceuticals*.

[20] Telekes A, Hegedus M, Chae CH, Vékey K (2009). Avemar (wheat germ extract) in cancer prevention and treatment. *Nutrition and cancer*.

[21] Boros LG, Cascante M, Lee WNP (2002). Metabolic profiling of cell growth and death in cancer: applications in drug discovery. *Drug Discovery Today*.

[22] Boros LG, Lapis K, Szende B (2001). Wheat germ extract decreases glucose uptake and RNA ribose formation but increases fatty acid synthesis in MIA pancreatic adenocarcinoma cells. *Pancreas*.

[23] Comín-Anduix B, Boros LG, Marin S (2002). Fermented wheat germ extract inhibits glycolysis/pentose cycle enzymes and induces apoptosis through poly(ADP-ribose) polymerase activation in Jurkat T-cell leukemia tumor cells. *The Journal of Biological Chemistry*.

[24] Judson PL, Al Sawah E, Marchion DC (2012). Characterizing the efficacy of fermented wheat germ extract against ovarian cancer and defining the genomic basis of its activity. *International Journal of Gynecological Cancer*.

[25] Marcsek Z, Kocsis Z, Jakab M, Szende B, Tompa A (2004). The efficacy of tamoxifen in estrogen receptor-positive breast cancer cells is enhanced by a medical nutriment. *Cancer Biotherapy and Radiopharmaceuticals*.

[26] Chou TC (2006). Theoretical basis, experimental design, and computerized simulation of synergism and antagonism in drug combination studies. *Pharmacology Review*.

[27] Tai CJ, Wang CK, Chang YJ, Lin CS (2012). Aqueous extract of *Solanum nigrum* leaf activates autophagic cell death and enhances docetaxel-nduced cytotoxicity in human endometrial carcinoma cells. *Evidence-Based Complementary and Alternative Medicine*.

[28] Tai CJ, Wang CK, Tai CJ (2013). Aqueous extract of *Solanum nigrum* leaves induces autophagy and enhances cytotoxicity of cisplatin, doxorubicin, docetaxel, and 5-fluorouracil in human colorectal carcinoma cells. *Evidence-Based Complementary and Alternative Medicine*.

[29] Wang P, Henning SM, Heber D (2010). Limitations of MTT and MTS-based assays for measurement of antiproliferative activity of green tea polyphenols. *PLoS ONE*.

[30] Ongena K, Das C, Smith JL, Gil S, Johnston G (2010). Determining cell number during cell culture using the scepter cell counter. *Journal of Visualized Experiments*.

[31] Berridge MV, Tan AS (1993). Characterization of the cellular reduction of 3-(4,5-dimethylthiazol-2-yl)-2,5-diphenyltetrazolium bromide (MTT): subcellular localization, substrate dependence, and involvement of mitochondrial electron transport in MTT reduction. *Archives of Biochemistry and Biophysics*.

[32] Tung JN, Cheng YW, Hsu CH (2011). Normoxically overexpressed hypoxia inducible factor 1-alpha is involved in arsenic trioxide resistance acquisition in hepatocellular carcinoma. *Annals of Surgical Oncology*.

[33] Shintani T, Klionsky DJ (2004). Autophagy in health and disease: a double-edged sword. *Science*.

[34] Mueller T, Voigt W (2011). Fermented wheat germ extract—nutritional supplement or anticancer drug?. *Nutrition Journal*.

